# Supplementary material for: Effect of Multiple Rounds of Enrichment on Metabolite Accumulation and Microbiota Composition of Pit Mud for Baijiu Fermentation
Source: Foods. 2023 Apr 9;12(8):1594. doi: 10.3390/foods12081594 (PMC10137600; doi:10.3390/foods12081594)
Supplement: Supplementary file 1 [file foods-12-01594-s001.zip › foods-2304843-figure S1.pdf]

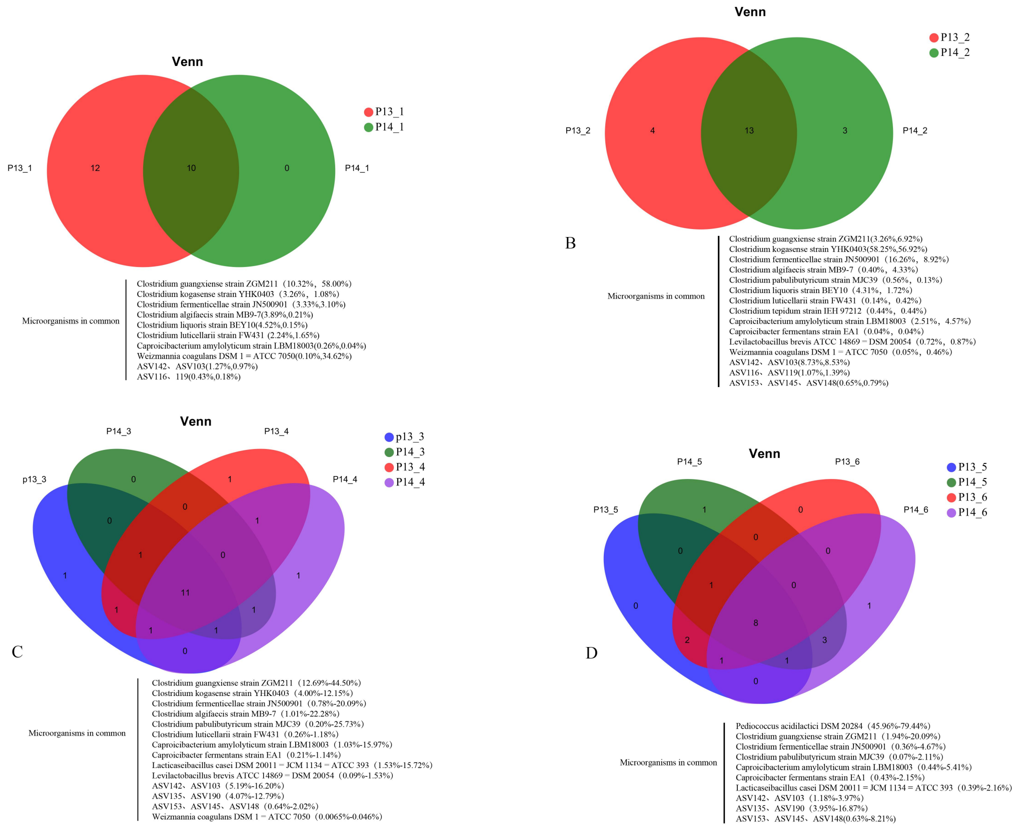

Figure S1 Venn diagram of species at different enrichment stages.(A)During the first round of enrichment, P13-1 and P14-1 co-owned species.(B)Adaptation period, P13-2 and P14-2 co-owned species.(C)During the Main fermentation period, P13-3,P13-4,P14-3 and P14-4 co-owned species.(D)Fermentation anaphase, P13-5、P13-6、P14-5 and P14-6 co-owned species.
