# Supplementary material for: Effect of Multiple Rounds of Enrichment on Metabolite Accumulation and Microbiota Composition of Pit Mud for Baijiu Fermentation
Source: Foods. 2023 Apr 9;12(8):1594. doi: 10.3390/foods12081594 (PMC10137600; doi:10.3390/foods12081594)
Supplement: Supplementary file 1 [file foods-12-01594-s001.zip › Table S1.pdf]

Table S1 Statistical analysis of sequencing data of amplicon library of EFB bacteria 16S rDNA

Sample	SequencesNumber	Asvs	Chao1	Shannon	Coverage
P13_1	55861	322	320.67	3.37	0.9997
P13_2	58762	62	62.00	2.38	1.0000
P13_3	68658	73	72.00	2.87	1.0000
P13_4	61615	52	52.20	2.04	0.9999
P13_5	54423	54	54.00	1.38	1.0000
P13_6	56794	56	56.00	2.49	1.0000
P14_1	56999	39	39.00	1.48	1.0000
P14_2	60307	57	57.00	2.40	1.0000
P14_3	67120	52	52.00	2.84	1.0000
P14_4	55696	45	45.00	2.36	1.0000
P14_5	48510	36	36.00	1.16	1.0000
P14_6	64485	35	35.00	1.07	1.0000
